# IgA Targeting Human Immunodeficiency Virus-1 Envelope gp41 Triggers Antibody-Dependent Cellular Cytotoxicity Cross-Clade and Cooperates with gp41-Specific IgG to Increase Cell Lysis

**DOI:** 10.3389/fimmu.2018.00244

**Published:** 2018-03-29

**Authors:** Maxence Duchemin, Marwa Khamassi, Lin Xu, Daniela Tudor, Morgane Bomsel

**Affiliations:** ^1^Laboratory of Mucosal Entry of HIV-1 and Mucosal Immunity, Department of Infection, Immunity and Inflammation, Cochin Institute, CNRS UMR 8104, Paris, France; ^2^INSERM U1016, Paris, France; ^3^Université Paris Descartes, Sorbonne Paris Cité, Paris, France

**Keywords:** antibody-dependent cellular cytotoxicity, IgA, human immunodeficiency virus-1, human immunodeficiency virus envelope protein gp41, mucosal immune system

## Abstract

The protective efficacy of human immunodeficiency virus-1 (HIV-1) antibodies (Abs) remains mostly correlated with their *in vitro* neutralizing activity engaging their Fab region. However, anti-HIV-1 Abs also mediate a broad array of Fc-mediated effector functions including Ab-dependent cellular cytotoxicity (ADCC), which depend primarily on the Ab isotype. While ADCC is commonly associated with HIV-1 gp120 envelope-specific IgGs, whether IgAs, especially those targeting the HIV-1 gp41 envelope, also mediate ADCC remains elusive. Therefore, to assess the capacity of IgA specific for HIV-1 to induce Fcα-mediated ADCC, we used the gp41 envelope-specific IgA transformed from the broadly neutralizing 2F5-IgG we have previously reported to induce ADCC. We demonstrate that 2F5-IgA engages FcαRI (CD89), expressed on human monocytes used as effector cells, to induce the lysis of HIV-1 Clade A- and B-infected target cells by ADCC. Furthermore, the 2F5-IgA and 2F5-IgG cooperate to enhance target cells lysis by ADCC. Cooperation in ADCC is also observed between 2F5-IgA and the broadly neutralizing 10E8-IgG. These results provide a new perspective for IgA in protection against HIV-1 acquisition or reservoir eradication and suggest that inducing IgA by vaccination, in particular when targeting gp41, in combination with IgG could strengthen protection by complementary and cooperative activities with IgG.

## Introduction

The protective efficacy of antibodies (Abs) to human immunodeficiency virus-1 (HIV-1) remains mainly correlated with their *in vitro* neutralizing activity. However, Abs targeting the virus can also mediate a broad array of Fc-mediated effector functions for clearing viral particles and infected cells, irrespective of their neutralizing activities (1). One of the most relevant Fc-mediated function is Ab-dependent cellular cytotoxicity (ADCC) engaging natural killer (NK) cells, monocytes, macrophages, or neutrophils as innate effector cells (2). Hence, ADCC is one of the earliest IgG function identified in HIV-infected individuals (3). Furthermore, we and others showed that in HIV-infected subjects who spontaneously control viral replication, including Elite Controllers and Long-Term Slow Progressors, ADCC-inducing IgGs increased in correlation with reduction of AIDS progression (4, 5) and exhibit great breadth in ADCC responses (6). IgG-mediating ADCC, frequently detected in breast milk, correlates with lack of mother-to-child HIV-1 transmission (7). Furthermore, in highly exposed but persistently seronegative individuals, IgG that preferentially recognized Env in its CD4-bound conformation can also mediate ADCC (8).

Antibody-dependent cellular cytotoxicity activity remains predominantly studied for non-neutralizing (9) or neutralizing (10) IgG targeting gp120, the most variable subunit of the HIV-1-envelope. Likewise, V1/V2-gp120-specific IgG with ADCC activities were induced in the RV144 clinical vaccine trial and correlated with reduced risk of infection (11, 12). In contrast, ADCC potential of gp41-specific IgG remains understudied, despite extensive studies on the ADCC capacity of the non-neutralizing gp41-specific IgG 98.6, targeting the membrane proximal external region (MPER) of the gp41 HIV-1-envelope subunit or a conformational epitope (13).

Fc-mediated Ab functions are remarkably complex and depend on Ab isotype, subclass, degree of glycosylation, and on the distribution of isotype-specific Fc receptors (FcRs) on effector cells (14). Hence, like IgG, IgA interacts with effector cell surface-expressed FcRs, the best known one being the Fc alpha RI (FcαRI/CD89). In turn, IgA mediates a panel of innate immune responses including not only ADCC but also phagocytosis and cytokine synthesis (15).

The role of IgA in HIV-1 target cell lysis by ADCC remains elusive, except one study suggesting an ADCC potential for anti-gp120 IgA (16) and none questioning the ADCC activity of IgA targeting gp41. Moreover, in the RV144 HIV-1 vaccine trial, vaccine-induced anti-gp120 IgAs have been proposed to compete with anti-gp120 IgGs, thereby reducing IgG-mediated ADCC effector function (17, 18), indicative of a greater affinity of IgA than of IgG for gp120. Importantly in these studies, ADCC effector cells lacked FcαRI/CD89 expression, and therefore intrinsic Fcα-dependent ADCC activities of IgA could not be evaluated; neither could potential synergy of the two isotypes.

Therefore, understanding respective IgG and IgA antiviral functions and focusing on gp41-specific Abs remain key issues for the design of an HIV-1 vaccine. Accordingly, we previously showed that a prophylactic HIV-1 vaccine based on gp41-conserved MPER subunits induces gp41-specific IgG and IgA that were both correlated with full protection against mucosal SHIV-1 infection in non-human primates (19). In vaccinated animals, protection correlated with gp41-specific IgG capable of ADCC. However, in this instance, the intrinsic gp41-specific IgA ADCC activity was not evaluated.

Genetic engineering that allows for comparison of IgG and IgA functions by IgG and IgA isotype switching revealed striking different biological properties between switched isotypes (20). We have previously studied isotype influence using as a model the broadly neutralizing anti-HIV Ab 2F5. We demonstrated that, while containing identical variable regions, 2F5-IgA2 and 2F5-IgG1 have distinctive affinities, fine three-dimensional epitope specificities for HIV-1 gp41-MPER, and antiviral functions (21). Although the 2F5-IgG is the best example of a gp41-neutralizing Ab able to utilize the FcγRI to achieve *in vitro* ADCC activity in addition to its neutralization potential, as we have shown earlier (9), whether IgA also mediates Fc-mediated effector functions remains unknown.

Until now, studies on anti-HIV-1 immunity including those exploring ADCC have mainly focused on the Clade B subtype widespread in Europe and the United States, but responsible for less than 15% of infections worldwide. Despite recent studies on immune responses against Clade C virus that is widespread in Asia and Africa, immunity induced by Clade A virus largely present in Eastern Europe and Africa remains understudied, although these two latter clades account for almost 60% of HIV-1 infections worldwide (22).

Here, we investigated whether the 2F5 Ab under IgA isotype engages FcαRI/CD89-expressing effector cells to lyse HIV-1^+^ target cell by cross-clade ADCC.

## Results

Antibody-dependent cellular cytotoxicity is a mechanism whereby an Ab bridges effector and target cells, triggering the release of lytic enzymes that lyse the target cell. Cell bridging results from binding of the Ab Fc-domain to its cognate FcR at effector cell surface and from specific recognition by the Ab Fab-domain of its antigen on target cells.

### 2F5-IgA and 2F5-IgG Bind to Effector Cells

To investigate the ADCC potential of 2F5-IgA2 (referred to below as 2F5-IgA) and permit comparison with 2F5-IgG1 (referred to below as 2F5-IgG), we used human primary monocytes as effector cells that express both FcαRI/CD89 and FcγRI/CD64, therefore capable of binding both IgA and IgG, and also secrete granzymes and perforins for target cell lysis (23, 24). Hence, in addition to expressing FcγRI that binds IgG1 and IgG3 and act as a potent effector cell to induce ADCC (25–27) and Ab-dependent cell phagocytosis (ADCP) (28), myeloid cells including monocytes express the FcαRI that binds to all IgA forms, monomeric or dimeric, and equally to isotype 1 and 2 (29). Accordingly, by using specific Abs and quantification by flow cytometry, we found that >85% of the effector monocytes expressed CD89/FcαRI or CD64/FcγRI. In addition, using anti-IgA2 as secondary Ab, we found that 2F5-IgA (2.5 µg/ml) bound to FcαRI on 26.5 ± 0.14% monocytes, whereas using anti-kappa light chain detection to allow for a direct comparison between isotypes, 2F5-IgA and 2F5-IgG (2.5 µg/ml) bound to FcαRI and FcγRI on 5.0 ± 0.4% and 5.0 ± 0.8% human monocytes, respectively (Table 1).

**Table 1:** P1 Clade A and B-specific 2F5-IgA and IgG binding to effector and target cells.

			2F5 antibodies (μg/ml)	Specific labeling (positive cells%)			
				Anti-human kappa light chain		Anti-human IgA	Anti-human IgG
	
				IgA	IgG	IgA	IgG
Target cells	CEM-Nkr coated with	P1-A	2.5	19.0 ± 0.6	59.0 ± 5.8	86.0 ± 2	94.3 ± 1.4
		P1-B	2.5	34.0 ± 1.6	39.0 ± 3.6	88.0 ± 1.3	91.3 ± 0.9
		P1-C	2.5	1.7 ± 0.1	1.0 ± 0.1	Not tested (nt)	nt

	Infected	Clade B (JR-CSF)	2.5	18.9 ± 1.4	7.2 ± 0.5	16.5 ± 1.2	17.2 ± 1.5
		Clade A (92UG031)	2.5	13.1 ± 2.9	10.6 ± 2.4	12.3 ± 2.8	8.2 ± 1.9

Effector cells		Monocytes	2.5	5.0 ± 0.4	5.0 ± 0.8	26.5 ± 0.14^a^	16 ± 0.95^b^

*2F5-IgA and 2F5-IgG were incubated with purified monocytes as effector cells, or P1 Clade A-, B-, and C-coated cells, as target cells, or uncoated cells as negative control for 1 h at 4°C. Alternatively, antibodies were incubated with human immunodeficiency virus-1 (HIV-1) Clade B- or Clade A- infected cells or uninfected cells as negative control for 45 min at room temperature (RT). Irrelevant IgA and IgG were used as negative controls. Specific binding was detected using either mouse fluorescein isothiocyanate (FITC)-conjugated anti-human kappa light chain to allow for direct comparison of both 2F5 isotypes or either FITC- or allophycocyanin (APC)-conjugated anti-human IgA/IgG and analyzed by flow cytometry, as indicated in Section “Materials and Methods.” Values represent the percentage of monocytes or P1-coated cells that stain positive for each antibody ± SEM, and the percentage of mAb^+^ cells representing a distinct cell population among infected (Gag^+^ or GFP^+^) cells ± SEM (for HIV-1 Clade B- and Clade A-infected target cells, respectively) relative to the percentage of HIV-1-infected cells. Binding of 2F5-IgA to uncoated and uninfected cells was negligible. Binding of irrelevant IgA and IgG to P1-coated cells were negligible (0.4%) and to infected cells, it was fixed at 1% and substracted from the specific binding values shown in the table. *n* = 5 independent experiments*.

*^a^Binding evaluated using an anti-IgA2 as a secondary antibody*.

*^b^Binding evaluated using an anti-IgG as a secondary antibody (9)*.

### 2F5-IgA and 2F5-IgG Bind to HIV-1 Clade A- and B-Infected Target Cells

As target cells, we first used either CD4^+^T CEM cells that are resistant to NK cells (CEM-NK^r^)—infected with HIV-1 clade B (JR-CSF), as we previously reported when characterizing the ADCC potential of 2F5-IgG (9), or green fluorescent protein (GFP)-reporter CD4^+^ T-cells infected with HIV-1 Clade A (92UGO31). To allow direct comparison of target cell recognition by the two 2F5 isotypes, specific binding was detected using either mouse anti-human kappa light chain or alternatively anti-human IgA or IgG and quantified by flow cytometry (Table 1). Irrelevant IgA and IgG were included as negative controls. For the same 2F5 Ab concentration (2.5 µg/ml), 2F5-IgA bound HIV-1 Clade B-infected cells in higher number than 2F5-IgG (18.9 ± 1.4% versus 7.2 ± 0.5%) when detected with anti-human kappa light chain, whereas binding was similar (16.5 ± 1.21% versus 17.2 ± 1.5%) when revealed with anti-human IgA or IgG. Using higher concentration (10 µg/ml) did not increase specific binding efficiency. Similarly, 2F5-IgA and 2F5-IgG bound to 13.1 ± 2.9% and 10.6 ± 2.4% HIV-1 Clade A-infected cells as when detected with anti-human kappa light chain, similar values being detected with anti-human IgA or IgG (Table 1), respectively. Such low HIV-1-infected cell binding values for MPER-specific Abs correspond to those reported by others (10). In this later study as in the results reported here, Ab concentrations needed to lyse cells by ADCC are much lower than those used to measure Ab binding, as often the case with functional tests.

### 2F5-IgA and 2F5-IgG Bind to Target Cells Coated with HIV-1 Clade A and B gp41 Peptides

We characterized earlier the P1 peptide (aa 649–684) as an extended gp41-MPER peptide encompassing the canonical 2F5 epitope (ELDKWA on gp41 Clade B) (30). P1 allows for HIV-1 binding to its mucosal receptor galactosylceramide and mediates HIV-1 transcytosis across epithelial cells and uptake by dendritic cells (30). Furthermore, we reported earlier that the structures of the 2F5-specific epitope when complexed to 2F5-IgG and within P1, as we defined by NMR, are similar, which is not the case within shorter MPER fragments (31). Thus, P1 appears as an optimized gp41-derived subunit mimicking the MPER conformation within HIV envelope and suitable to be used as a vaccine immunogen in order to induce HIV-blocking Abs. Hence, we also used P1 peptide as a gp41-derived immunogen in a clinical vaccine strategy in humans (32) that allowed full protection against viral challenges in the monkey. Furthermore, *in vivo* protection in monkey correlated with P1-specific IgG capable of mediating ADCC (19). We thus used HIV-1 envelope subunit P1 derived from HIV-1 Clade A (P1-A), Clade B (P1-B), and Clade C (P1-C) to coat CEM-NK^r^ cells as an alternative target cell model, a procedure commonly used in ADCC studies (9, 19, 33–35).

**Figure 1:**
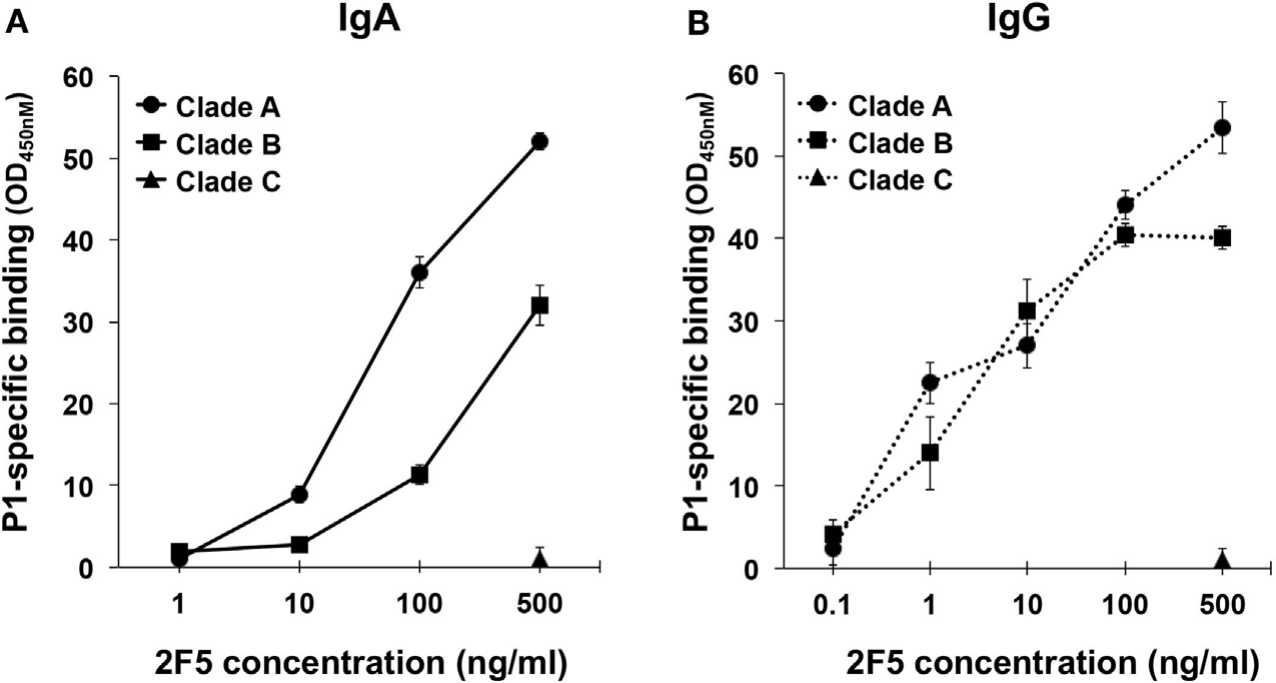
2F5-IgA and 2F5-IgG bind to P1 Clades A and B in a dose-dependent manner. The specificity of **(A)** 2F5-IgA (solid line) and **(B)** 2F5-IgG (dotted line) for P1 Clade B (square), Clade A (circle), and Clade C (triangle) was evaluated by ELISA. Mouse anti-human kappa light chain was used for direct comparison of both 2F5 isotypes. Specific binding (OD, 450 nm) is plotted as a function of 2F5 antibody (Ab) concentration (ng/ml). No binding was detected for the irrelevant IgA or IgG used as negative controls (OD 450 nm: <0.1). Values represent mean ± SEM, derived from three independent experiments performed in duplicate.

First, we characterized the interaction of 2F5-IgA and 2F5-IgG with P1-A, P1-B, and P1-C using ELISA. We have previously shown that 2F5-IgA and 2F5-IgG bind to P1-B (21). Here, we found that both 2F5-IgA and 2F5-IgG specifically bound to P1-B and P1-A dose dependently, but not to P1-C (Figures 1A,B). 2F5-IgA bound P1-B more efficiently than 2F5-IgG, but the latter bound P1-A slightly better than 2F5-IgA. 2F5-IgA did not recognize P1-C as expected, since the 2F5-IgG canonical epitope ELDKWA in Clade B gp41 is mutated to ALDSWK in Clade C gp41, a motif not recognized by 2F5-IgG (36).

Next, CEM-NK^r^ target cells were coated with P1-A, P1-B, or P1-C, and specific recognition by 2F5-IgA and 2F5-IgG was measured using flow cytometry (Table 1). 2F5-IgA or 2F5-IgG (2.5 µg/ml) bound P1-coated cells more efficiently than infected target cells. Hence, 19.0 ± 0.6% and 59.0 ± 5.8% of P1-A-coated cells and 34.0 ± 1.6% and 39.0 ± 3.6% of P1-B-coated cells stained positive for 2F5-IgA and 2F5-IgG, respectively, when detected with anti-human kappa light chain. As expected, neither 2F5-IgA nor 2F5-IgG bound P1-C-coated cells, thus serving as a negative control. By using anti-human IgG or IgA for detection, 86.0 ± 2% of P1-A and 88.0 ± 1.3% of P1-B-coated cells stained positive for 2F5-IgA, while >90% stained positive for 2F5-IgG independent of the clade and again 2F5 isotypes failed to bind P1-C-coated cells.

Altogether, HIV-1 Clade A- and B-infected cells, as well as P1-coated cells, constitute suitable target cells to assess ADCC mediated by 2F5-IgA and 2F5-IgG.

### 2F5-IgA Triggers the Lysis of HIV-1-Infected Cells by ADCC Cross-Clade

**Figure 2:**
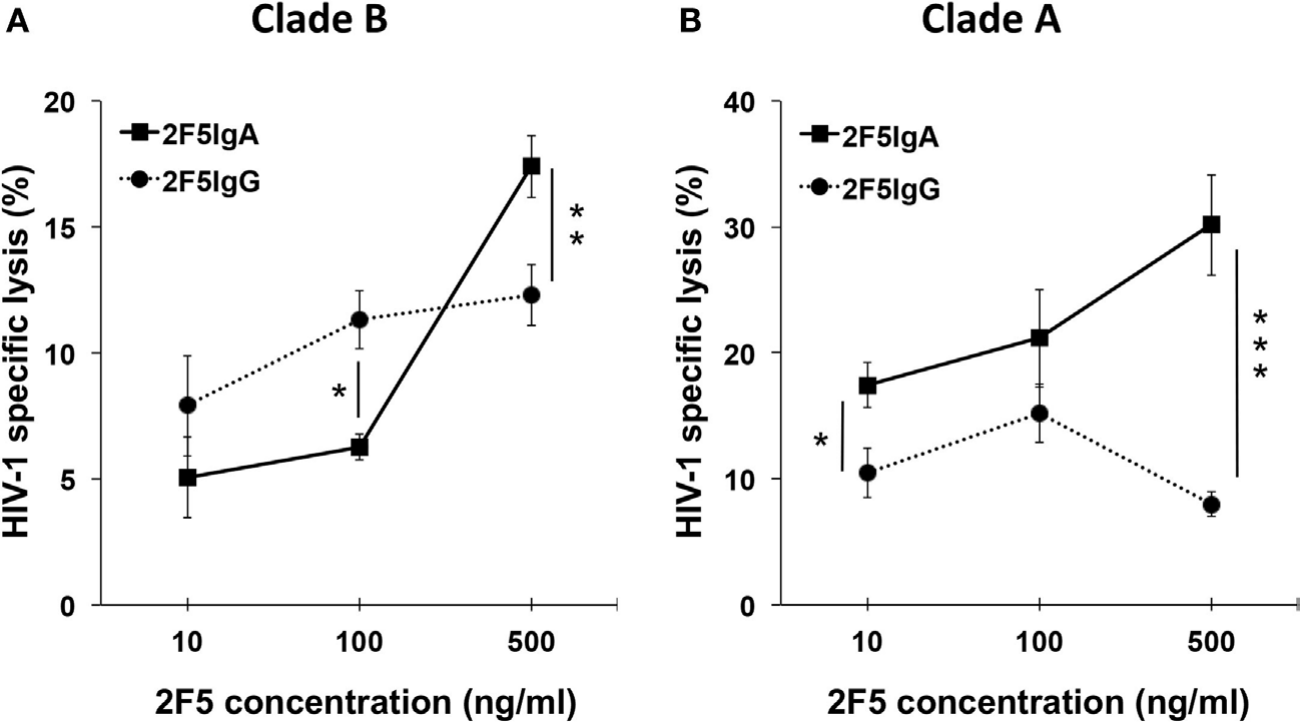
Human immunodeficiency virus-1 (HIV-1)-specific antibody-dependent cellular cytotoxicity (ADCC) triggered by 2F5-IgA compared to 2F5-IgG. Target CD4^+^ T CEM-NK^r^ cells infected with **(A)** HIV-1 Clade B (JR-CSF) or **(B)** green fluorescent protein (GFP)-reporter CD4^high^ CCR5^high^ T-cell lines infected with HIV-1 Clade A (92UG031) were stained with PKH26 and then incubated with the indicated concentrations of 2F5-IgA or 2F5-IgG for 30 min at room temperature (RT), prior to addition of effector monocytes (effector:target ratio, 10:1). After 4 h, the percent of infected living (Gag^+^ or GFP^+^) target cells was measured by flow cytometry. The percent of ADCC was calculated using the formula: 100 × (% of infected/PKH26^+^ target cells without antibody − % of infected/PKH26^+^ target cells with antibody)/(% of infected/PKH26^+^ target cells without antibody). Values represent means of HIV-1-infected target cell-specific lysis ± SEM, from six independent experiments performed in duplicate or triplicate for each clade, *p < 0.05, **p < 0.02, and ***p < 0.005, unpaired Student’s *t*-test of 2F5-IgA versus 2F5-IgG-mediated ADCC.

We first evaluated the capacity of 2F5-IgA to mediate lysis of HIV-1 R5-tropic Clade B- and Clade A-infected target lymphocytes by ADCC. As initially transmitted viruses are mainly R5-tropic, such target cells might mimic cells infected early in disease transmission. Target cells were surface labeled with PKH26 prior to incubation first with Abs and second with effector cells. The ADCC potential of 2F5-IgA was assessed by calculating the percentage of infected living (Gag^+^ or GFP^+^) target cells and calculated according to the formula reported in Section “Materials and Methods.” The Ab concentration required for ADCC is usually much lower than that used for neutralization of HIV-1 cell infection, as we previously showed for 2F5-IgG (9). Thus, concentrations of 2F5-IgA and IgG as low as 10–500 ng/ml were next used to initiate ADCC. 2F5-IgA triggered lysis by ADCC of target cells infected by both clade of HIV-1, namely A and B, in a concentration-dependent manner, reaching 16.0 ± 1.3% lysis for Clade B (Figure 2A) and 30.1 ± 4.05% lysis of Clade A (Figure 2B). As a negative control, irrelevant IgA or IgG induced only a low background lysis of target cells (<2–3%, respectively). Increasing Ab concentration to 1.5 µg/ml did not increase target cell lysis (data not shown), suggesting that 2F5-IgA binding to target cells was saturated at 500 ng/ml. Furthermore, 2F5-IgG not only induced ADCC of Clade B-infected target cells (Figure 2A) as we showed earlier (9) but also induced ADCC of Clade A-infected target cells (Figure 2B). At 500 ng/ml, 2F5-IgA induced ADCC of both Clade B and Clade A-infected target cells with higher efficiency than 2F5-IgG (Clade B: 16.0 ± 1.3% versus 9.0 ± 2%, unpaired *t*-test *p* < 0.05; Clade A: 30.1 ± 4.05% versus 7.86 ± 0.8%, unpaired *t*-test *p* < 0.006; Figures 2A,B). In contrast, and as expected, HIV-1 Clade C-infected cells were not lysed by ADCC by either 2F5 isotypes (data not shown) due to the lack of binding to the MPER region as shown above (Figure 1), and confirming the specificity of HIV-1 Clade A- and B-infected target cell lysis.

Altogether, 2F5-IgA, more efficiently than 2F5-IgG, promotes HIV-1 Clade A and B-infected cells lysis by ADCC.

### 2F5-IgA Has the Potential to Induce the Lysis of P1 Clade A- and Clade B-Coated Target Cells by ADCC

**Figure 3:**
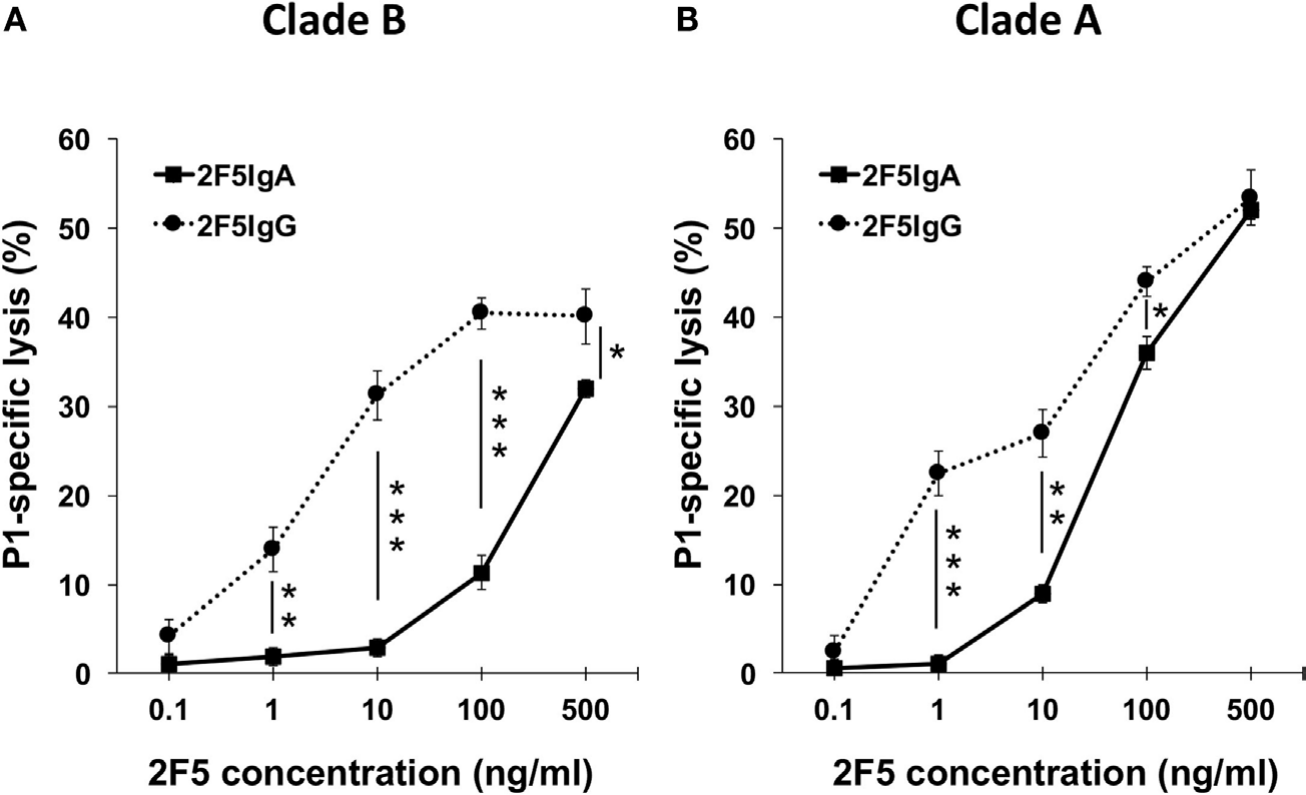
2F5-IgA and 2F5-IgG induce the lysis of P1-A- and P1-B-coated cells in a dose-dependent manner. The efficacy of 2F5-IgA (solid line, square) and 2F5-IgG (dotted line, circle) to induce the lysis of P1-B **(A)**, and P1-A coated cells **(B)** was evaluated by flow cytometry. Target P1 Clade A and B cells were double stained with PKH26 and carboxyfluorescein diacetate succinimidyl ester and then incubated with the indicated concentrations of 2F5-IgA or 2F5-IgG for 30 min at room temperature (RT), prior to addition of effector monocytes (effector/target ratio: 10:1). Specific lysis determined as we reported (9) is plotted as a function of 2F5 antibody (Ab) concentration (ng/ml). Values represent mean ± SEM, showing the percentage of specific Ab-dependent cellular cytotoxicity (ADCC) killing derived from five independent experiments performed in duplicate. *p < 0.05, **p < 0.02, and ***p < 0.005, unpaired Student’s *t*-test of 2F5-IgA versus 2F5-IgG mediated ADCC.

To analyze the mechanism by which 2F5-IgA induced ADCC, we next used P1-coated target cells that are easier to prepare in quantities compared to HIV-1-infected cells. To measure ADCC, P1-coated target cells were double labeled with PKH26 and carboxyfluorescein diacetate succinimidyl ester (CFSE) prior to incubation with Abs followed by addition of effector cells, as we reported (9). By using the gating strategy described in supplementary Fig. 1, lysed target cells (membrane PKH26^+^, cytosolic CSFE^−^) are then easily distinguished from live target (membrane PKH26^+^, cytosolic CSFE^+^) and non-labeled effector cells. A dose-dependent ADCC-mediated lysis of P1-B and P1-A-coated cells was observed for 2F5-IgA and 2F5-IgG. At the highest 2F5 concentration (500 ng/ml), 2F5-IgA induced 32.0 ± 2.51% of P1-B-coated cell lysis, whereas 2F5-IgG-mediated 40.1 ± 1.41% of P1-B-coated cell lysis; using P1-A-coated cells as targets, 2F5-IgA induced 52.0 ± 3.1% target cell lysis, similar to 2F5-IgG that induced 53.0 ± 1% of target cell lysis (Figures 3A,B). As a negative control, irrelevant IgA or IgG induced only a low background lysis of target cells (<4–5%, respectively). In confirmation of ADCC specificity, both 2F5-IgA and 2F5-IgG were unable to bind P1-C (Table 1) and failed to induce P1-C-coated target cell lysis (P1-C-coated cell-specific lysis, <0.5%). P1-A-coated cells lysis by 2F5-IgA was higher compared to that of P1-B-coated cells for all concentrations tested (Figures 3A,B), whereas no significant differences between P1-A and P1-B-coated cell lysis was induced by 2F5-IgG (Figures 3A,B). Taking into account the respective efficiency of 2F5-IgA and 2F5-IgG to bind to P1-A and P1-B-coated cells (Table 1), 2F5-IgA-mediated ADCC of P1-A-coated target cells was the most robust compared to other conditions. Overall, the magnitude of P1-coated lymphocytes lysis correlated with the 2F5 isotype binding efficiency to target cells reported in (Table 1) (Pearson *r* = 0.8150, *p* < 0.05).

Altogether, 2F5-IgA mediates specifically ADCC of P1-A- and P1-B-, but not P1-C-coated target cells, and the magnitude of lysis was higher on P1-A-coated cells.

### The ADCC Activity of 2F5-IgA or 2F5-IgG Depends on FcαRI or FcγRI

**Figure 4:**
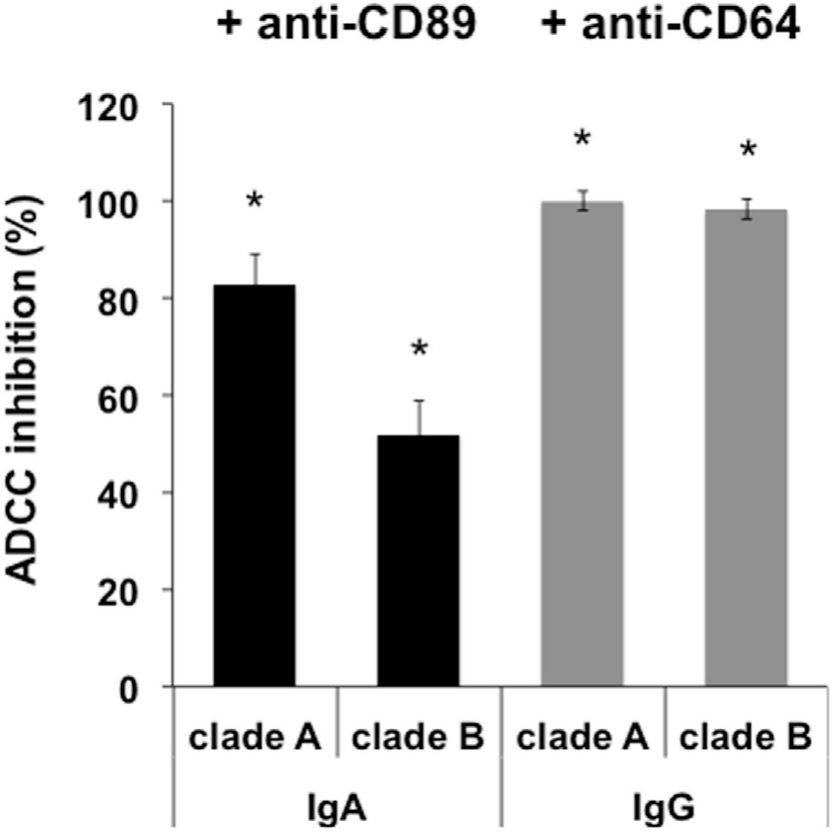
Antibody-dependent cellular cytotoxicity (ADCC) depends on FcαRI and FcγRI. Monocytes were preincubated or not with either anti-CD89- or anti-CD64-blocking antibody for 1 h at 37°C before incubation with double-stained CD4^+^ T CEM-NK^r^ cells coated with P1. Target cell lysis was evaluated as shown in Figure 3. Values represent the percent of ADCC-induced cell lysis reduction in the presence of monocytes preincubated with anti-CD89 or anti-CD64 antibody. Values represent mean inhibition ± SEM. *p < 0.005, unpaired Student’s *t*-test compared to ADCC in the absence of antibodies.

Next, engagement of isotype-specific FcR in ADCC was evaluated by preincubation of monocytes with anti-human CD89 Ab. Inhibition with anti-CD89 antibody resulted in a strong decrease of P1-A- and P1-B-coated target cell lysis triggered by 2F5-IgA (83.0 ± 6% and 52.0 ± 7%, respectively; Figure 4). ADCC of both P1-A and P1-B mediated by 2F5-IgG was fully inhibited by preincubation with anti-CD64 Ab (Figure 4).

Furthermore, 2F5-IgA binding to effector cells was required for triggering ADCC, as preincubation of monocytes with a 100-fold excess (50 µg) of irrelevant IgA together with 2F5-IgA (500 ng/ml) resulted in a 91.0 ± 5% reduction of ADCC induced by 2F5-IgA alone.

Thus, IgA-mediated ADCC depends on FcαRI signaling and not on alternative Fcα/μR (37). Similarly, IgG-induced ADCC depends on FcγRI signaling, as we have already shown (9).

### 2F5-IgG Potentiates 2F5-IgA-Mediated Lysis of P1-A Target Cells by ADCC

2F5-IgA and IgG have different epitope affinity and specificity (21), suggesting that the two isotypes could simultaneously bind to target cells, as we reported for HIV-1-infected Langerhans cells (21). Furthermore, as monocyte effector cells express both FcαRI and FcγRI, they can bind 2F5-IgA and 2F5-IgG simultaneously. We therefore evaluated whether 2F5-IgA and 2F5-IgG could mediate in concert the lysis of P1-coated target cells by ADCC.

**Figure 5:**
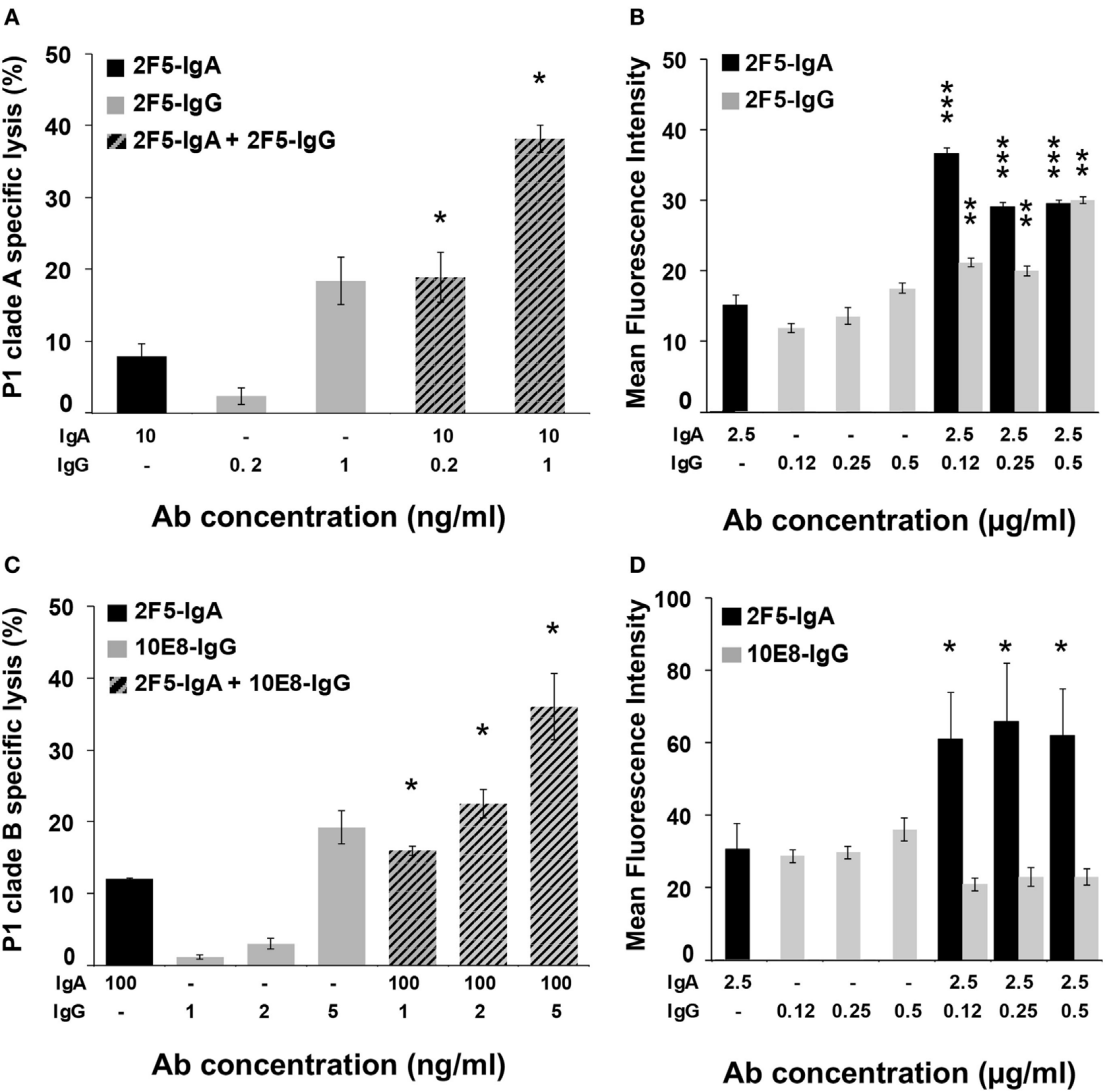
2F5-IgA cooperates with 2F5-IgG to induce lysis of P1-A target cells by antibody-dependent cellular cytotoxicity (ADCC) as a result from increased binding to target cells. **(A,C)** CD4^+^ T CEM-NK^r^ cells coated with P1-A and P1-B and double stained with PKH26/carboxyfluorescein diacetate succinimidyl ester were incubated with either 2F5-IgA (black bars), 2F5-IgG or 10E8-IgG (gray bars), or with a combination of both, at indicated concentrations, for 30 min at room temperature (RT). Then, effector cells were added at an effector/target ratio of 10:1. Hatched bars represent the experimental values ± SEM of ADCC induced by the combination of 2F5-IgA and 2F5-IgG or 10E8-IgG at indicated concentrations. *n* = 3 independent experiments performed in duplicate. *p < 0.05, Student’s *t*-test between the arithmetical sum of cell lysis induced by each isotype separately and cell lysis induced by both isotype together. **(B,D)** CD4^+^ T CEM-NK^r^ cells coated with P1-A and P1-B were incubated with 2F5-IgA (black bars), 2F5-IgG or 10E8-IgG (gray bars), or with a combination of both at indicated concentrations for 1 h at 4°C. Specific binding was detected using either mouse fluorescein isothiocyanate (FITC)-conjugated anti-human IgA or allophycocyanin (APC)-conjugated anti-human IgG, or the combination of both, and analyzed by flow cytometry, as indicated in the Section “Materials and Methods.” Values represent MFI of specific binding. *n* = 5 independent experiments. ***p < 0.0001 and *p* < 0.0002, **p < 0.001, Student’s *t*-test between MFI values of the binding of IgA or IgG alone with that of IgA or IgG in the combination mix, respectively.

Thus, 10 ng of 2F5-IgA together with 0.2 or 1 ng of 2F5-IgG were incubated with P1-A-coated target cells, and ADCC was monitored after effector cell addition. In parallel, ADCC induced by each isotype alone at the same concentration was evaluated (Figure 5A). The addition of 2F5-IgG to 2F5-IgA increased target cell lysis compared to 2F5-IgA alone. Furthermore, lysis magnitude induced by combining the two 2F5 isotypes was always significantly higher than the arithmetic sum of lysis induced by each isotype alone at the same concentration (for 10 ng/ml 2F5-IgA and 0.2 ng/ml 2F5-IgG: 18.9 ± 3.5% versus 10 ± 2%, Student’s *t*-test *p* < 0.05; and for 10 ng/ml 2F5-IgA and 1 ng/ml 2F5-IgG: 38.2 ± 1.9% versus 25.4 ± 3.8%, Student’s *t*-test *p* < 0.05). Thus, 2F5-IgA and 2F5-IgG act in cooperation to lyse P1-A target cells by ADCC. We next evaluated whether the cooperation of the two isotypes in mediating ADCC was the result of increased P1-A target cells binding by each isotype in the presence of the other compared to binding by each isotype when incubated alone with target cells (Figure 5B). Accordingly, simultaneous incubation of P1-A target cells with 2F5-IgA (2.5 µg/ml) and increasing concentration of 2F5-IgG (from 0.12 to 0.5 µg/ml) compared to incubation with each isotype alone resulted in increased target cell binding at all concentrations tested. Of note, increase was higher at the lowest IgG concentration [MFI of 2F5-IgA binding alone versus in the presence of 2F5-IgG (0.125 μg/ml): 16.8 ± 1.1 versus 37.2 ± 0.7, Student’s *t*-test *p* < 0.001; MFI of 2F5-IgG (0.125 µg/ml) binding alone versus in the presence of 2F5-IgA: 11.5 ± 0.4 versus 21.3 ± 0.4, Student’s *t*-test *p* < 0.01]. In contrast, no cooperation was detected when P1-B-coated target cells were used, most likely due to more efficient binding of 2F5-IgA for P1-B relative to P1-A (Table 1), resulting in steric hindrance thus reducing 2F5-IgG access to P1-B-coated cells.

We then evaluated the cooperative activity of 2F5-IgA with 10E8-IgG, another broadly neutralizing Ab specific for a gp41 epitope contiguous to the 2F5 one and included in the P1 peptide. In this case again, 2F5-IgA (100 ng/ml) cooperated with increasing doses of 10E8-IgG (from 1 to 5 ng/ml) to induce an efficient P1 Clade-B-target cells lysis at all concentrations tested (Figure 5C). Lysis magnitude induced by combining the two isotypes was significantly higher than the arithmetic sum of lysis induced by each isotype alone at the same concentration (for 100 ng/ml 2F5-IgA and 1 ng/ml 10E8-IgG: 16 ± 0.7% versus 13 ± 0.29%, Student’s *t*-test *p* < 0.05; for 100 ng/ml 2F5-IgA and 2 ng/ml 10E8-IgG alone: 22.5 ± 2% versus 16 ± 0.31%, Student’s *t*-test *p* < 0.05; and for 100 ng/ml 2F5-IgA and 5 ng/ml 10E8-IgG alone: 36 ± 4.6% versus 31 ± 2.3%, Student’s *t*-test *p* < 0.05). As shown above for 2F5-IgA and 2F5-IgG, cooperation of 2F5-IgA and 10E8-IgG (0.125–0.5 µg/ml) in increasing target cell lysis directly correlated with an increase in target cell binding (Figure 5D). However, in this case, only 2F5-IgA binding increased (MFI of 2F5-IgA (2.5 µg/ml) alone versus in the presence of 10E8-IgG (0.125 μg/ml): 31.8 ± 3.1 binding IgA versus 60.2 ± 5.7, Student’s *t*-test *p* < 0.1).

## Discussion

This study is the first rigorous examination of the ability of an anti-HIV-1 envelope IgA to induce the lysis of target cells by ADCC cross-clade.

Worldwide, HIV-1 infection occurs predominantly *via* sexual intercourse at mucosal sites. It is thus critical to evaluate whether mechanisms additional to neutralization could block this transmission pathway. IgA is the prevalent Ab isotype at mucosal sites. Furthermore, FcαRI/CD89 was identified on phagocytes located in mucosal tissues (25) and monocytes that, along with macrophages and neutrophils, are the predominant FcR bearing cells in the cervicovaginal mucosa (38). The present characterization is in line with the new vision concerning the importance of Ab isotype profile in HIV-1 vaccine efficacy and protection in mucosal challenge, such as Ab-mediated internalization of HIV-1 virions that was shown to differ among Ab isotypes (28). Here, we hypothesized that anti-HIV-1 envelope IgA, including neutralizing ones, alone or in cooperation with IgG, could induce destruction of infected cells by ADCC using monocytes expressing both FcγR and FcαR as effector cells.

2F5-IgA was used for three main reasons: first because 2F5-IgA offers a model of a neutralizing gp41-specific IgA for which no Fc-mediated antiviral functions, including ADCC, have been studied. Thus, evaluation of the ADCC potential of a NAb IgA will provide information concerning the contribution of the Fc region of gp41-specific IgA in the protection against HIV-1; second, as we reported earlier using P1-coated cells and HIV-1 infected cells as targets, 2F5-IgG elicits ADCC in a mechanism strictly dependent on binding to ELDKWA, the canonical 2F5 epitope on Clade B HIV-1 exposed on the target cell (9), and on FcγRI expressed on monocytes served as effector cells; third, this evaluation complements the comparative analysis of anti-HIV-1 functional properties of two isotypes, IgA and IgG, of the same Ab 2F5. Hence, we previously demonstrated (21) that, although harboring the same VH regions and light chain as 2F5-IgG, 2F5-IgA has increased antigen affinity for gp41 and HIV-1 viruses due to a difference in fine epitope specificities and increased Fab-dependent antiviral activities against Clade B HIV-1.

Here, we demonstrate for the first time that 2F5-IgA can trigger ADCC of target cells present at different steps of HIV infection (39). Hence, HIV-1 envelope subunit-coated target cells, an experimental model frequently used in ADCC studies by us (9, 19, 30–32) and others (33–35), could recapitulate the initial step in infection, when viruses bind to target cells exposing specific epitopes poorly accessible at the free virus surface (40). Alternatively, HIV-1-infected cells represent targets once infection is established, although in this case, CD4 downregulation and Tetherin/BST-2 antagonism could reduce HIV envelope ADCC-mediating epitope exposure (41).

In addition, our results provide a comprehensive comparative analysis of the 2F5-IgA and 2F5-IgG revealing additional isotype-specific links between defined Fc and antigen-binding domains. Although much is known about the role of variable domains in the neutralization breadth and potency of 2F5 as IgA and IgG, the contribution of Fc-domains to their activities is, by contrast, poorly characterized for 2F5-IgG and has never been characterized for IgA.

Indeed, *in vivo*, the Ab antiviral efficiency is highly dependent not only on the Fab–antigen interactions that block viral entry but also on interaction of the Ab Fc-domain with its cognate FcR expressed on the innate effector cells (37). By combining the action of the Fab and Fc regions, Abs mediate a broad array of Fc-dependent effector functions including ADCC, Ab-dependent virus inhibition (ADCVI), and ADCP, in addition to neutralization.

Remarkably complex, Fc-mediated functions link the specific adaptive immune system to the powerful effector functions of the innate immune system and depend critically on Ab isotype. Effector functions of IgA could differ from the ones of IgG due to differences in Fc-affinity for corresponding FcαR versus FcγR and the lack of IgA binding by the C1q component of complement. Furthermore, Ab binding to FcR is not sufficient for ADCC to occur, but also requires triggering of effector cell degranulation. Accordingly, IgA mediates ADCC by monocytes, but in contrast to IgG, and although IgA binds to NK cells, FcαR stimulation by IgA failed to trigger ADCC on NK cells [data not shown and (15)].

Here, the profile of 2F5-IgA and 2F5-IgG binding to their respective FcR, FcαRI, and FcγRI was similar (Table 1) and had no impact in triggering ADCC. Rather, the target antigen, P1-A versus P1-B, influences the extent of ADCC. A positive correlation between isotype binding to target cells and isotype-specific ADCC was observed for both P1 clades. Likewise, ADCC of infected cells mediated by 2F5-IgA and 2F5-IgG appeared less efficient than that of P1-coated target cells, in direct association with differences in target cell binding (Table 1). These differences correspond to a limited exposure of the Ab cognate epitopes at the infected cell surface, a likely consequence of incomplete or incorrectly folded envelope protein at cell surface expression commonly reported on infected cells (42), compared to P1-coated cells. In addition, during the 4 h of the assay, infected cells do release free viral particles to which Abs could also bind, in turn limiting the Ab availability for target cell binding and subsequent ADCC. This suggests that additional Fc-mediated functions such as phagocytosis of IgA or IgG opsonized viral particles by the effector cell might compete with 2F5-IgA and 2F5-IgG-dependent ADCC, leading to a lower percentage of infected cell lysis.

Of note, 2F5-IgA mediates HIV-1-infected cell lysis more efficiently than IgG, whereas the opposite is observed for P1-coated cells. These differences are in good correlation with the target cell binding efficiencies shown in Table 1 reporting that 2F5-IgG binds better to P1-coated cell than 2F5-IgA, whereas 2F5-IgA binding to HIV-infected cells is superior compared to 2F5-IgG. Therefore, one likely explanation for the difference in ADCC-mediated lysis hierarchy between the two isotypes is the difference in isotypes binding to ADCC target cells.

Clade A is widely distributed worldwide, and the number of Clade A-infected individuals exceeds that infected by Clade B. However, Clade A HIV-1 is poorly studied. The MPER region targeted by 2F5 is well conserved in A and B clades, unlike in Clade C, and equally recognized by the 2F5 isotypes. Therefore, the cross-clade ADCC mediated by 2F5-IgA and 2F5-IgG against P1-B and P1-A-coated target cells constitutes an important antiviral mechanism beyond neutralization that warrant attention in antiviral strategies such as vaccines or passive immunotherapies.

Human immunodeficiency virus-1 infection largely occurs through mucosal epithelium, and persistent reservoirs likely form and hide in mucosal tissues (43). IgAs are the first line of defense at mucosal surfaces. Mucosal IgA might thus induce the lysis of target cells, either prior to infection when the virus is surface bound or when actually CD89 infected (39), by ADCC through engagement of the Fcα receptor. All forms of IgA have been shown to bind FcαRI/CD89 (44), suggesting that our results may also apply to dimeric IgA2, the main IgA isotype at mucosal sites as well as to dimeric IgA1. FcαRI/CD89 is identified not only on a variety of cell types within the immune system but also on phagocytes located in mucosal areas where IgA is the prevalent Ab isotype (43, 45). Accordingly, serum and secretory IgA Abs from HIV-infected individuals can lyse X4 HIV-1-infected cells, although in this study epitope specificity was not studied (46). Our present results thus provide a new perspective on the role of IgA in protection against mucosal HIV-1 acquisition, by suggesting that the induction of IgA by vaccination, in particular targeting gp41, might strengthen protection by complementary and combined activities with IgG.

Similar levels of IgA and IgG are detected at the vaginal mucosa, and IgA is quantitatively the second most abundant class of Ab in the blood. The concurrent antiviral functions of IgA and IgG against HIV-1 have been poorly studied. Early studies on the mucosal immune response in a non-HIV context reported that the addition of secretory IgA to IgG increased IgG-dependent ADCC mediated by monocytes, the two isotypes acting in cooperation (47). We now demonstrate that concerning HIV-1, 2F5-IgA cooperates not only with 2F5-IgG but also with 10E8-IgG, another broadly neutralizing gp41-specific Ab, to increase cell lysis by ADCC. In both cases, 2F5-IgA binding to target cells is increased in the presence of the IgG, either 2F5 or 10E8. Improved cell lysis could thus result from the recognition of one target cell by the two different isotypes. In turn, both isotypes either bind to different effector cells or alternatively to a single one expressing both FcαR and FcγR. The RV144 clinical trial analysis also addressed the interference of IgA- in IgG-mediated ADCC. There, vaccine-raised serum gp120-specific IgA blocked gp120-specific IgG-mediated gp120-coated cell lysis ADCC, most likely due to higher affinity of the IgA than the IgG for gp120 (17). Several although not exhaustive reasons can be proposed to explain this apparent discrepancy with the cooperation between isotypes we report here. First, in their ADCC model, Tomaras et al. used effector cells that did not expressed the FcαRI and were thus unable to lyse target cell mediated by IgA, although IgA bound efficiently target cells and displaced IgG binding. Thus, one cannot exclude that if effector cell would have expressed the FcαRI, this high affinity IgA would have increased IgG-mediated ADCC rather than just decreasing it due to IgG displacement (17). Second, concentrations of Abs used in Tomaras’ study are 10- to 100-fold higher than that used in the experiments reported here. This difference could participate in the divergent results of the two studies. Third, the capacity of the gp120 epitopes targeted by the RV144 vaccine-induced Abs in mediating ADCC differs from that of the gp41 epitopes, suggesting that the epitopes targeted by the Ab is of chief importance for isotype cooperation in ADCC (48). Fourth, divergences observed could also result from differences in Ab glycosylation as IgG, but not IgA, glycosylation pattern has been shown to impact FcR binding/affinity and antibody-mediated cell function (49, 50).

Nevertheless, the cooperation in ADCC between isotypes targeting gp41 we report here is in line with the cooperation in blocking HIV-1 transfer from LCs to T-cells that we observed previously (21), and with *in vivo* studies reporting on the cooperation between anti-HIV-1 gp120-IgG and corresponding IgA obtained by genetic engineering that when infused in monkey, completely protected animals against intrarectal virulent SHIV challenge (51), whereas each Ab alone resulted in partial protection.

In conclusion, our present study clearly demonstrates that not only 2F5-IgG is provided with an ADCC potential but also that IgA, such as gp41-specific 2F5-IgA, can induce the lysis of target cells by ADCC cross-clade. Our study emphasizes the critical value of inducing IgA and IgG Abs in the mucosa with complementary and cooperative activities by vaccination. Both isotypes together could combine suboptimal defenses at the mucosal and systemic levels to completely prevent virus acquisition (51).

Furthermore, similar to the ADCC-inducing Abs that develop earlier in HIV-1-infected patients than do neutralizing Abs (3), additional antiviral activities involving the Fcα and γ region of Abs that are induced by vaccination may also appear earlier after contact of the host with the virus. Hence, inducing both IgA and IgG by vaccination and assessing vaccine efficacy by measuring IgA and IgG Fc-mediated functions emerge as a crucial goal in antiviral strategies.

## Materials and Methods

### Monoclonal Abs and Peptides

2F5-IgA2 (2F5-IgA) and IgG1 (2F5-IgG) were obtained as previously described (21). Non-specific IgA and IgG controls were from The Binding Site Group Ltd. (Birmingham, UK) and Jackson ImmunoResearch Europe Ltd. (Suffolk, CB8 7SY, UK), respectively. Peptide P1 (aa 650–685) derived from gp41 envelope subunit of Clade B (P1-B) was from HXB2 HIV-1: SQTQQEKNEQELLELDKWASLWNWFDITNWLWYIK (30), of Clade A (P1-A) was from 99UGA07072 HIV-1: SQIQQKKNEQDLLALDKWANLWNW-FDISNWLWYIR, and of Clade C (P1-C) was from Bw96Bw0502 HIV-1: SQTQQEKNEQ-ELLALDSWKNLWNWFSITNWLWYIK. Peptides were chemically synthesized [purity > 95%, Biopeptide (LA, USA) for P1-B and United BioSystems (VA, USA) for P1-A and P1-C].

### HIV-1 Viruses

Viruses were from the NIH AIDS Reagent Program: the HIV-1 JR-CSF (clade B, R5-tropic) expression plasmid was used for JR-CSF HIV-1 virus stock production by transfection of 293T cells as previously described (52), whereas the HIV-1 primary isolate 92UG031 (clade A, R5) was amplified on peripheral blood mononuclear cells (PBMCs), as previously described (21).

### Effector Cells

Primary human monocytes were purified from healthy donor PBMC by negative selection using human monocyte enrichment kits (StemCell Technologies, France). For each experiment, a pool of PBMCs from three different donors were used to purify monocytes to limit donor variability. Resulting monocytes were FcγRIII negative (data not shown).

### Target Cells

Human CD4^+^T CEM-NK-resistant (CEM-NK^r^) lymphocytic cells line expressing CCR5 were obtained from the NIH AIDS Reagent Program. The CD4^high^ CCR5^high^ GFP-reporter T-cell line (53) was a kind gift from Dr O. Kutsch (The University of Alabama at Birmingham). To prepare infected target cells for ADCC, 3 × 10^6^ CEM-NK^r^ lymphocytes were infected with 800 ng p24 of JR-CSF and GFP-reporter cells with 600 ng p24 of HIV-1 clade A (92UG031) for 2 h at 37°C and further cultured for 40 h. Infection was monitored by intracellular p24 detection as described in Ref. (52) or by monitoring GFP fluorescence by flow cytometry resulting in 40–50% infected (p24^+^) viable cells for Clade B and 8–20% infected (GFP^+^) for Clade A, an infection level required to obtain consistent ADCC levels.

Alternatively, CEM-NK^r^ cells (1 × 10^6^ cells) were coated with P1-A, P1-B, or P1-C (2.5 µM) for 1 h at room temperature (RT) as described (9).

### Recognition of P1-A, P1-B, and P1-C by 2F5-IgA and 2F5-IgG

Evaluation of 2F5-IgA and 2F5-IgG binding to P1-A, P1-B, and P1-C peptides was performed by ELISA as described (21) by coating microtiter plates (Peptide Immobilizer, Exiqon) with appropriated P1 (100 ng/well) overnight at 4°C in PBS. Comparative Ab binding at indicated concentrations was detected using a biotinylated mouse anti-human Ig kappa light chains (BD Pharmingen) followed by streptavidin-HRP.

### Ab Binding to Target Cells

For detection of HIV-1 envelope on HIV-1-infected target cells, or P1 on P1-coated target cells, 2F5-IgA or 2F5-IgG (2.5 µg/ml) were incubated with 10^5^ target cells prepared, as described above, for 1 h at 4°C or 45 min at RT, respectively, followed by either fluorescein isothiocyanate (FITC)-conjugated goat anti-human IgA or IgG (Jackson ImmunoResearch, West Grove, PA, USA) at 10 µg/ml, allophycocyanin-conjugated goat anti-human IgA or donkey anti-human IgG (Jackson ImmunoResearch, West Grove, PA, USA) or FITC-conjugated mouse anti-human kappa light chain (BD Biosciences, San Jose, CA, USA), as indicated by the manufacturer. Alternatively when indicated, anti-IgA2 [The Binding Site Group Ltd. (Birmingham, UK)] was used as secondary Ab. In the case of infected target, cells were next fixed with 4% paraformaldehyde, and HIV-1 clade B-infected cells were further stained for intracellular Gag with the phycoerythrin (PE)-coupled anti-Gag KC57 murine monoclonal Ab (Beckman Coulter GmbH), whereas GFP fluorescence was used to monitor HIV-1 clade A infection in GFP-reporter target cells.

Antibody binding to target cells was quantified by flow cytometry using a Guava EasyCyte flow cytometer (Merck-Millipore) and analyzed using the dedicated InCyte software.

### Ab Binding to Effector Cells

The specificity of 2F5-IgA and 2F5-IgG binding to monocytes FcαR or FcγR was evaluated by incubation of 2.5 µg/ml Abs with 10^5^ monocytes for 1 h at 4°C, followed by either FITC-conjugated mouse anti-human kappa light chain or alternatively by anti-human IgA2 or anti-human IgG-FITC as mentioned above.

Antibody binding to effector cells was quantified by flow cytometry using a Guava EasyCyte flow cytometer (Merck-Millipore) and analyzed using the dedicated InCyte software.

### Phenotyping FcR on Monocytes

For FcαR or FcγR detection on effector cells, 10^5^ freshly isolated monocytes were incubated with PE-mouse IgG1k anti-human FcαRI (CD89) or with FITC mouse IgG1k anti-human FcγRI (CD64) mAbs, as indicated by the manufacturer (BD Biosciences, USA). Specific labeling was quantified by flow cytometry using a Guava EasyCyte (Merck-Millipore) and analyzed using the dedicated InCyte software.

### ADCC Assays

(i) *ADCC using infected cells as targets*: ADCC was determined using an adaptation from Ref. (10). Briefly, HIV-1-infected cells were labeled with the membrane dye PKH26 (Sigma-Aldrich, St. Louis, MO, USA) as recommended by the manufacturers and incubated in triplicated wells with 2F5-IgA and 2F5-IgG at indicated concentrations or the irrelevant IgA2 or IgG1 for 30 min at RT. Effector monocytes were then added at a 10:1 effector/target ratio. Plates were spun 1 min at 300 *g* to promote cell contacts and further incubated for 4 h at 37°C in a 5% CO_2_. Co-cultures were then fixed with 4% paraformaldehyde. Then, HIV-1 Clade B-infected CEM-NK^r^ were stained for intracellular Gag with the FITC-coupled anti-Gag KC57 murine monoclonal Ab (Beckman Coulter GmbH), whereas GFP expression was used to quantify HIV-1 Clade A-infected target cells. Cells were immediately acquired on a Guava easyCyte flow cytometer (Merck-Millipore), and the frequencies of Gag^+^ or GFP^+^ cells among PKH26^+^ cells were determined. ADCC is calculated using the following formula: 100× (% of Gag^+^ or GFP^+^/PKH26^+^ target cells without Ab − % of Gag^+^ or GFP^+^/PKH26^+^ target cells with Ab)/(% of Gag^+^ or GFP^+^/PKH26^+^ target cells without Ab). Irrelevant IgA or IgG Abs gave a background ADCC of 3 and 2%, respectively.
(ii) *ADCC using P1-coated cells as targets*: ADCC was determined using an adaptation from Ref. (54), as previously described for 2F5-IgG (9). Briefly, after coating with different P1 peptides, cells were dually labeled with the membrane dye PKH26 and the viability dye 5-(and dye-6-) CFSE (Molecular Probes, Eugene, OR, USA) as recommended by the manufactures, before incubation with 2F5-IgA, 2F5-IgG, or the irrelevant IgA or IgG for 30 min at RT. Effector Cells were then added, at a 10:1 effector/target ratio; plates were spun 1 min at 300 *g* to promote cell contacts, and further incubated for 4 h at 37°C. Flow cytometry dot plot of dual-stained target cells incubated in the same conditions as the effector-target co-cultures were used to set the gate of living double-positive target cells, in which the cell membrane is still intact. Target cell lysis by monocytes was defined by the loss of CFSE but the retention of PKH26 resulting in the emergence of a lysed PKH26^+^CFSE^−^ population within the PKH26^+^ gate (i.e., % of CFSE^−^ cells within PKH26 high gate), as we described (9). Irrelevant IgA or IgG Abs at 500 ng/ml (the highest concentration) gave a background ADCC of 4 and 3%, respectively.

### FcR Blockade

When indicated, FcRs CD89 or CD64 were blocked by preincubation of monocytes effector cells with a blocking anti-human CD89 monoclonal Ab (MIP7c) (Abcam, France) or a blocking anti-human CD64 monoclonal Ab (BD Pharmingen, USA) at 10 µg/ml at 37°C for 1 h prior to being mixed with ADCC-inducing Abs and target cells. Percentage blocking is calculated using the formula 100× (% of ADCC with blocking/% ADCC without blocking).

### Statistical Analysis

Statistical significance was analyzed by the two-tailed Student’s *t*-test using Prism 5 (GraphPad, San Diego, CA, USA) software.

## Author Contributions

DT and MB conceived and designed the experiments. DT, MB, MD, MK, and LX performed the experiments. DT, MD, MK, LX, and MB analyzed the data. DT and MB wrote the paper.

## Conflict of Interest Statement

The authors declare that the research was conducted in the absence of any commercial or financial relationships that could be construed as a potential conflict of interest. The reviewer MB and handling Editor declared their shared affiliation.
