# Supplementary material for: IgA Targeting Human Immunodeficiency Virus-1 Envelope gp41 Triggers Antibody-Dependent Cellular Cytotoxicity Cross-Clade and Cooperates with gp41-Specific IgG to Increase Cell Lysis
Source: Front Immunol. 2018 Mar 29;9:244. doi: 10.3389/fimmu.2018.00244 (PMC5884934; doi:10.3389/fimmu.2018.00244)
Supplement: Supplementary file 1 [file Data_Sheet_1.PDF]

"IgA targeting HIV-1 envelope gp41 triggers antibody-dependent cell cytotoxicity cross-clade and cooperates with gp41 specific IgG to increase cell lysis"

Duchemin et al.

Supplementary material and Figure 1

Gating strategy for the ADCC assay.

Target cells were double stained with PKH-26 and CFSE, then incubated with the indicated concentrations of the indicated antibody, here 2F5-IgA, for 30 min at RT, prior to addition of effector monocytes. After 4hrs of contact, ADCC was evaluated by flow cytometry.

We used the standard gating strategy on PKH26+ population established by Gomez-Roman et al (Gómez-Román et al., 2006) that quantifies CFSE loss in target cells when gated on PKH26+ cells compared to gating on target (PKH26+CFSE+) and effector cells (PKH26-CFSE-), thus identifying the emergence of CFSE-PKH26+, the cell population that is lyzed by ADCC.

Supplementary Figure 1 legend

A: Analysis of effector and target cells in the presence of 2F5-IgA.

Target and effector cells (upper left) were identified according to cell morphology by light-scatter parameters and gated on R1 for further analysis of both PKH26 and CFSE fluorescence parameters (dot plot of the gate R1, lower left). In this PKH26/CFSE dot plot, $PKH26^+$ target cells were gated in R2. Next (middle lower panel), within the R2 gate, $PKH26^+/CFSE^+$ live target cells defined the R3 gate and $PKH26^+/CFSE^-$ lyzed target cells, having lost CFSE as result of cell lysis, defined the R4 gate, whereas effector $PKH26^-$ cells were gated in R5. Back gating into forward scatter (FSC) and sideward scatter (SSC) plots (upper right) allowed to

visualize effector cells in pink, live target cells in yellow (partially overlapping with effector cells), and lyzed target cells in blue (partially overlapping with both effector and live target cells). Finally (lower right), ADCC is calculated as % cells in R4 / % total cell in R2 .

B: Analysis of effector and target cells in the absence of 2F5-IgA or in the presence of irrelevant IgA.

Target and effector cells are incubated as in A but in the absence of antibody or in the presence of irrelevant IgA. R3, R4, R5 gates (lower) as defined in A were back-gated into FSC/SSC plot (upper), allowing to visualize effector cells in pink and live target cells in yellow (partially overlapping with effector cells). Non specific lyzed cells in blue have also a scattered distribution overlapping with both effector and live target cells.

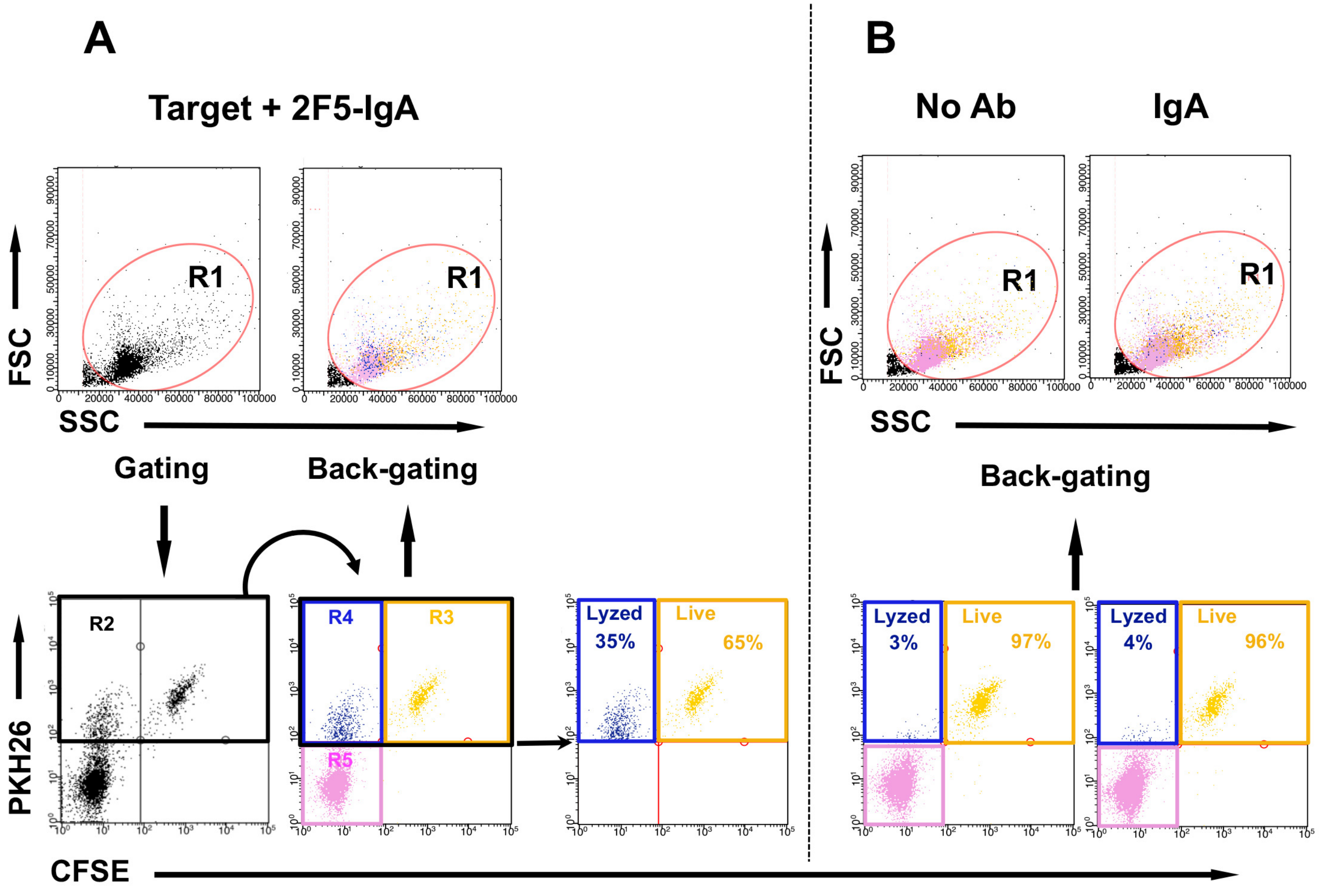

Supplementary Fig.1
